# Notices of New Publications

**Published:** 1846-06-01

**Authors:** 


### NOTICES OF NEW PUBLICATIONS.

*Lectures on the Nature and Treatment of Deformities, delivered at the Royal Orthorpædic Hospital, Bloomsbury Square. By* R. W. TAMPLIN, *Surgeon to the Hospital, etc., with numerous illustrations.* Philadelphia: Barrington & Haswell, 1846.

This is an octavo volume of 216 pages, issued as a part of "Bell's Select Medical Library," and we hail it as a valuable addition to the series, since the author is a sound, *practical* man, and in these lectures he gives us the result of a large experience in one of the best conducted and most valuable charities of London; a charity devoted exclusively to the treatment of Deformities.

Upon the yet *new* modes of relieving this class of surgical diseases, ( by the knife ) there is no lack of "writings," but for facts and well based principles there is room enough. Hear what the author promises:

"It is my intention, in the ensuing Lectures, to confine myself as much as possible to *facts; for facts alone* can be of service in the present condition of our knowledge of deformities." ( p. 11. )

True, the book is not occupied entirely nor mainly with a reiteration of cases. This is not indeed what we desire; in all such books of detail there is unavoidably much and useless repetition. What we need, is certain great principles, as a *result* of digested and analyzed facts, and a few of these facts as illustrations and confirmations of the principles. Such is the character of the work before us. For example:

"The principles laid down by Delpech, in his Orthomorphie, published at Paris in 1828, are comprised in the following rules:

1st. A tendon to be divided must not be exposed; and its division should be made by turning the instrument on one side, so that the line of incision may not be parallel to the division of the skin; without this precaution risk of exfoliation of the tendon is incurred.

2d. Immediately after division of the tendon, the divided ends should be brought into contact with each other, and kept in this position by a suitable apparatus during the entire period necessary for their union.

3d. Inasmuch as it can only take place by an intermediate fibrous substance, this substance, before it has become firm, can, and should be extended gradually and carefully, until it has assumed a degree of length equal to the shortened muscle.

4th. When this degree of extension has been effected, the parts should always be fixed in the position, and kept so until the new substance has acquired its requisite degree of consolidation.

"These, gentlemen," remarks our author, "are the principles upon which we are now acting, and from which we depart but in the slightest degree: they embody the entire docrine of the treatment of deformity, and have only to be followed out carefully to ensure success."

It ought to be understood, however, that 2d and 3d of these "principles" are yet in dispute, and that while such men as Delpech, (dec'd) Stromeyer, Stoess, Ammon, Samuel Cooper, Little and Liston contend for more or less delay in the application of extracting apparatus; Lorens, Sartorius, Scoutetten, Lenger, Duval, Bouvier, Velpeau, Fergusson and Detmold, advo cate, with certain qualifications, immediate extension.

Speaking of the section of the tendo-achilles, he remarks: (p. 35.)

"No risk is incurred beyond that appertaining to the slightest wound, and so morally and practically certain are its effects, that I have no chances of injury, no contingent evils to lay before you for investigation. Here we have divided some hundreds of tendo-achilles, and as yet we have experienced no unfavorable result!"

This assertion might seem like self-laudation and vain boasting to one who has known little of the operation, or, indeed, to one who has only met with those unfortunate illustrations of tenotomy with which the country abounds, which have been treated by men, often mere itinerating and irresponsible vagabonds, without judgment, science, experience, skill, or apparatus; the latter of which is in our estimation, in most cases, even more necessary than the knife, and requires also greater skill in its application: but to one who is accustomed to see the operation well done, on judiciously selected cases, and followed by careful and proper mechanical treatment, the success of the author is not surprising.

No space is allowed for a farther consideration of this excellent treatise, and we can only enumerate, in conclusion, the special deformities here considered under the general title.

Lecture second, third, fourth and fifth treat of the various forms of Talipes. Lecture sixth, seventh and eighth of deformities, &c., of the knee. Lecture ninth of the hip. Lecture tenth of Rachitis, in which are found some new and valuable contributions to its causes, pathology, varieties, &c. Lecture eleventh considers angular curvature of the spine, &c. Lecture twelfth and thirteenth of posterior and lateral curvature. Lecture fourteenth of Torticollis, contraction of lower jaw, shoulder joint, arm, forearm, wrist, toes, &c. &c.

F. H. H.

---

*Fordyce on Fever, second American Edition, with an Introduction, Philadelphia.* Ed. Barrington *and* Geo. Haswell, 1846; *pp.* 395, 8*vo.*

This is the quarterly publication for April, of the Select Medical Library, edited by John Bell, M. D., of Philadelphia. It is hardly necessary to commend this work to the attention of Students and Practitioners, since it is generaly admitted that in the description of the phenomena of fever,

Fordyce has not been excelled by any writer before or since his day. As a correct and graphic history, the work will never cease to be valuable, being in this respect independent of the mutability of doctrines. We can recall the gratification, which in former years we derived from two careful perusals of these dissertations, and we anticipate a renewal of the same enjoyment, when we can find leisure to give it a third, in the attractive form in which it is presented by this edition.

---

*Lectures on the operations of Surgery; and on Diseases and Accidents requiring operations, by* Robert Liston, Esq. F. R. S. *&c., with numerous additions by* Thomar D. Mutter, M. D. *&c.* Lea & Blanchard, *Philadelphia*, 1846: *pp.* 564, *8vo.*

The name of Liston is sufficient guaranty for the value of all that he may publish on operative Surgery, more especially as the American medical public are already familiar with two works by his hand, which have been re-published, and received with great approbation in this country. The present volume consists of lectures delivered in 1844, at the University College, London, which were afterward printed in the London Lancet. Prof. Mutter, however, has contributed more than half the amount of matter which the volume contains. While it is, perhaps, for the advantage of the American reader, to obtain in one volume the united labour of author and editor, we must say that we should have been better satisfied if Prof. M. had given his instructions in a separate form. The work is illustrated with numerous cuts, and, so far as we may judge from the examination we have been able to give to it, it is a valuable contribution to our stock of practical surgical treatise. Like all the publications which emanate from the prolific press of Lea & Blanchard, its typographical appearance is highly satisfactory. We may give it a more extended notice hereafter.

---

*A Clinical Introduction to the practice of Auscultation, and other modes of Physical Diagonsis; intended to simplify the Study of Diseases of the Lungs and Heart, by* H. M. Hughes, M. D., *Assistant Physician to Guy's Hospital, &c.*

Any work, the purpose or end of which is to promote simplification in the study and practice of physical exploration, deserves a cordial welcome. Such is one of the objects of this little treatise. A mistaken impression, that this subject is too recondite and difficult for general adoption, is the chief obstacle in the way of its availability by all who are willing to devote a reasonable amount of effort to qualify themselves for the varied responsibilities of Medical Practice. We have given this manual a perusal, and we commend it as, in our judgment, embracing nearly all that is necessary to be learned from books, respecting the physical signs of thoracic diseases We must, however, enter our protest against one statement of the author, viz., that satisfactory success is only attainable at public institutions. It is to be recollected that the author is one of the Physicians to Guy's Hospital, and that the book was prepared especially for pupils in the wards of that Institution. We do by no means wish to disparage the superiour advantages which are afforded by public institutions for becoming proficients in physical diagonsis; but we do mean distinctly to avow, that every practitioner, with good auditory organs, a respectable acquaintance with modern pathology, and good judgment, may, in a very short time, acquire a practical acquaintance with the subject, for all ordinary purposes, provided the attempt be resolutely made, with the belief that the attainment is as feasible as it is important.

---

*The Influence of Tropical Climates on European Constitutions, by* James Johnson, M. D. *etc; and* James Ranald Martin, Esq., *late Presiding Surgeon, and Surgeon to the Native Hospital, Calcuttta. From the Sixth London Edition, with notes by an American Physician.* New York, Samuel S. and William Wood. 8vo. 1846: pp. 624.

Of the comparatively few medical books, amongst the many with which the press teems, destined to survive the changes incident to progressive advancement in science, the celebrated work on Tropical Climates, by Dr. Johnson, the late distinguished editor of the Medico-Chirurgical Review, is a prominent example. Although relating to circumstances belonging to Tropical Climates, it abounds in observations which are applicable to every locality, and, hence, is by no means, of interest exclusively to those who inhabit the Torrid Zone. The peculiar ability and tact of Dr. Johnson as a writer, are sufficiently well known. Probably no writer in the medical profession of the present age has been so universally esteemed by medical readers. We can recall with vividness the interest with which we perused this work many years ago, and we shall avail ourselves of the earliest leisure to repeat the gratification.

It has been for a long time out of print, and the profession have reason to be under obligations to the Woods for its re-publication. The additions of Dr. Martin contain valuable information respecting the diseases of warm climates, and enhance the interest and usefulness of the work.

